# Stem11 score: toward rapid clinical prognostication for acute myeloid leukemia

**DOI:** 10.1016/j.exphem.2026.105441

**Published:** 2026-04-17

**Authors:** Tomoya Isobe, Manja Meggendorfer, Sebastian Wolf, Thomas Oellerich, Nicola K. Wilson, Berthold Göttgens

**Affiliations:** a Department of Hematology, Cambridge Stem Cell Institute, University of Cambridge, Cambridge, United Kingdom; b Center for Childhood Cancer Research, Children’ s Hospital of Philadelphia, Philadelphia, PA; c MLL Munich Leukemia Laboratory, Munich, Germany; d Department of Medicine II, Hematology and Oncology, University Hospital, Goethe University Frankfurt, Frankfurt am Main, Germany; e Frankfurt Cancer Institute (FCI), Frankfurt am Main, Germany; f German Cancer Consortium (DKTK), partner site Frankfurt/Mainz, a partnership between DKFZ and University Hospital Frankfurt, Germany

## Abstract

Acute myeloid leukemia (AML) is an aggressive form of myeloid malignancy with high relapse and poor survival rates. Despite recent advances in genomics-based risk classification, accurate prediction of patient outcomes remains a challenge, posing the need for complementary molecular information to enable precise treatment stratification. In the current study, we assessed the prognostic value of the recently developed 11-gene Stem11 signature in a uniformly treated cohort of 107 de novo AML patients and showed that Stem11 classification stratifies overall survival and response to allogeneic hematopoietic cell transplantation across the European LeukemiaNet risk groups. We further developed a NanoString-based Stem11 scoring system and validate its high concordance with RNA-sequencing-based scoring and its retained prognostic power to identify the most refractory AML subgroup. With its rapid turnaround time and standardized built-in analysis pipeline, our NanoString-based Stem11 scoring panel represents a faster yet reliable alternative to RNA sequencing, providing preclinical proof of concept for Stem11-based clinical decision support.

Acute myeloid leukemia (AML) is an aggressive hematological malignancy with a poor prognosis. Current genetic risk stratification remains insufficient for accurately predicting patient outcomes, and additional information that can refine and improve prospective risk prediction is urgently needed. Here, we developed a NanoString-based diagnostic assay measuring the prognostic Stem11 score. Our assay provided highly concordant Stem11 scores with RNA-sequencing-based quantification, thereby establishing a rapid and reliable transcriptional prognostication system for time-sensitive clinical decision support for AML. © 2026 The Author(s). Published by Elsevier Inc. on behalf of International Society for Experimental Hematology. This is an open access article under the CC BY license (http://creativecommons.org/licenses/by/4.0/)

Acute myeloid leukemia (AML) is a heterogeneous hematologic malignancy characterized by aberrant proliferation and differentiation arrest of myeloid progenitor cells. Although genomics-based classification has improved prospective survival prediction [1,2], considerable heterogeneity in clinical outcomes persists even within the established genetic risk groups [3], highlighting the need for additional molecular markers to further refine patient risk stratification.

Recent single-cell studies have revealed that heterogeneous myelo-monocytic differentiation hierarchies, ranging from stem-cell-like to mature monocyte-like blast populations, exist within individual patients across genetic subtypes and influence treatment response and survival [4,5]. Furthermore, activation of non-myeloid lineage programs has been associated with high-risk genetics [6] and disease relapse [7] in AML, suggesting prognostic relevance of capturing aberrant hematopoietic lineage programs expressed within individual patients. In alignment with this, we have recently characterized the mechanisms of differentiation perturbation in preleukemic mouse models using single-cell RNA sequencing (RNA-seq) and developed Stem11, a gene expression-based scoring system that complements cytogenetics- and mutation-based risk classifications to identify the highest risk patients with AML with transcriptional lineage ambiguity [8]. To enable rapid clinical decision support, in the current study, we present the development of a clinically implementable Stem11 testing platform using the NanoString nCounter system.

## Methods

### Patient Samples

For RNA-seq analysis, gene expression data for 107 uniformly treated de novo AML patients were downloaded from the European Genome-Phenome Archive (EGAD00001008484) [9]. Out of the 107 patients, 39 patients were included for the NanoString assay based on sample availability and quality (Supplementary Table E1).

### Nanostring Custom Panel Design

As a rapid Stem11 scoring platform, a custom NanoString nCounter panel was designed. Alongside the Stem11 genes [8], the previously published LSC17 genes [10] were included as an established transcriptional prognostic system for AML. For data normalization, seven housekeeping genes were further included from a previous custom NanoString nCounter panel [11]. The full list of the 35 genes in our custom panel is provided in Supplementary Table E2.

### Nanostring Assay and Analysis

The mean expression of six synthetic positive control single-strand DNAs and seven housekeeping genes was used as a size factor for data normalization. The normalized expression values were then log-transformed for downstream analyses. The Stem11 score was calculated as the sum of the *Z*-scores of the Stem11 genes, and the Stem11-high group was defined as the top 15th percentile of the cohort, as previously defined [8]. The LSC17 score was calculated based on the formula defined in the original publication [10]. The LSC17-high group was defined as patients with scores above the cohort median, consistent with the original publication. Patient survival was estimated using the Kaplan-Meier method, and the difference was tested using the log-rank test using the R package survival. For multivariate analysis, a Cox proportional hazards regression model was used.

## Results And Discussion

**Figure 1:**
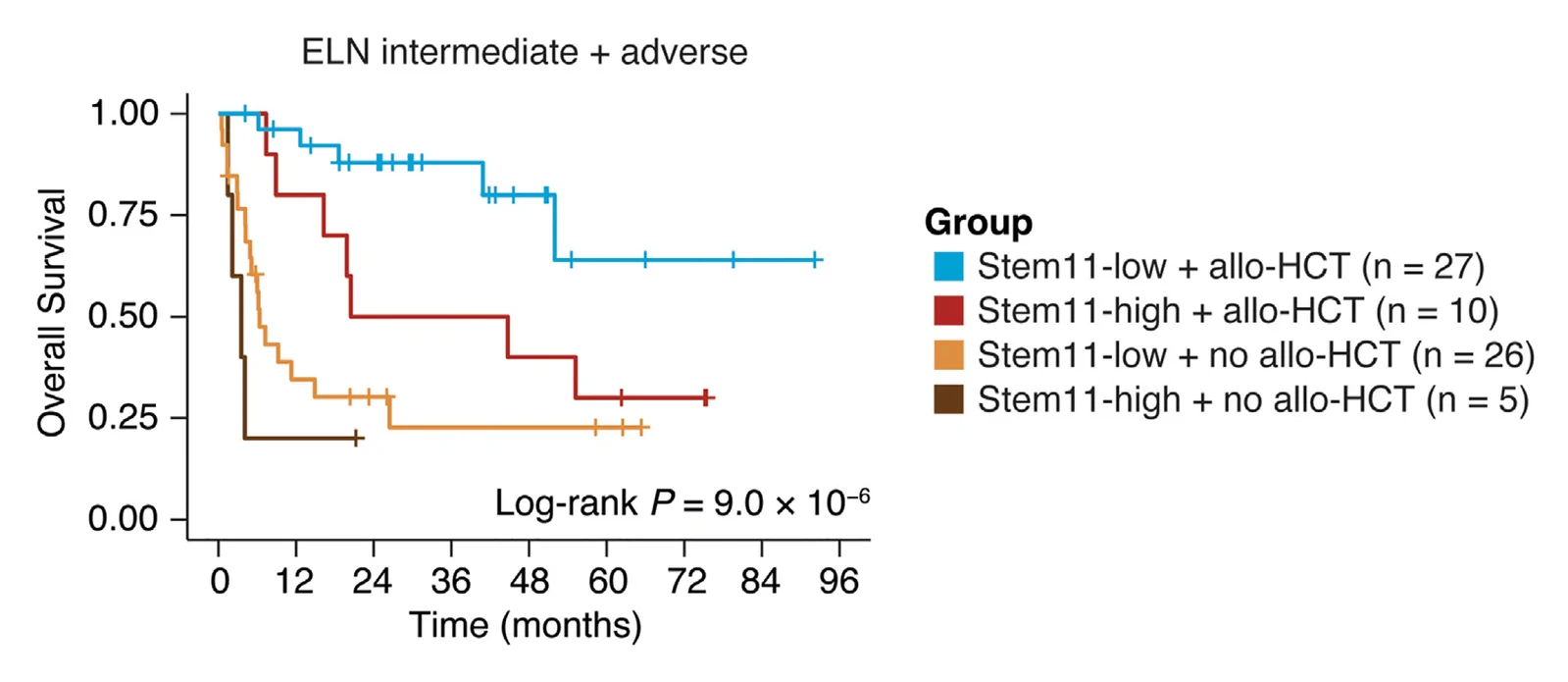
Prognostic significance of Stem11 in different treatment groups. Survival analysis comparing ELN-intermediate and adverse-risk patients stratified by Stem11 classification and HCT treatment. The *p* value is from the log-rank test.

In our previous study, we validated the prognostic value of the Stem11 scoring system using RNA-seq data from multiple independent AML cohorts, including both adult and pediatric patients [12 −14]. To further associate the Stem11-based classification with specific clinical decision support, we first analyzed a published RNA-seq dataset from de novo AML patients who all received uniform intensive chemotherapy with or without allogeneic hematopoietic cell transplantation (HCT) at a single university hospital in Germany [9]. In this cohort of 107 patients, we identified 16 patients with high Stem11 scores (hereafter, Stem11-high patients), including one favorable, six intermediate, and nine adverse-risk patients according to the European LeukemiaNet-2017 (ELN) classification [15]. Because HCT is considered for patients with ELN adverse-risk and intermediate-risk AML [15], we focused on survival differences by Stem11 classification and HCT status in these subgroups. Overall, Stem11-low patients benefited from HCT with a 5-year overall survival of 64.0% in contrast to 22.7% in Stem11-low patients treated without HCT (Figure 1). Although Stem11-high patients treated with HCT showed improved survival (median survival of 33 months with HCT vs. 3.5 months without HCT; Figure 1), post-transplant outcomes remained poor, with 5-year overall survival of 30.0%. The same trends were observed within the ELN adverse and intermediate-risk groups (Supplementary Figure E1A, B), and consistently, the Stem11 classification showed an independent prognostic value in a multivariate survival analysis including HCT status (Supplementary Figure E1C). This suboptimal HCT response highlights the need for novel therapeutic approaches for these highly refractory Stem11-high patients. Considering the immature transcriptional state captured using the Stem11 signature [8], the potential therapy may include venetoclax, which has shown efficacy in targeting primitive leukemic cells [16]. Collectively, these results demonstrate that the Stem11 classification can predict treatment response and identify patient subgroups most likely to benefit from HCT.

**Figure 2:**
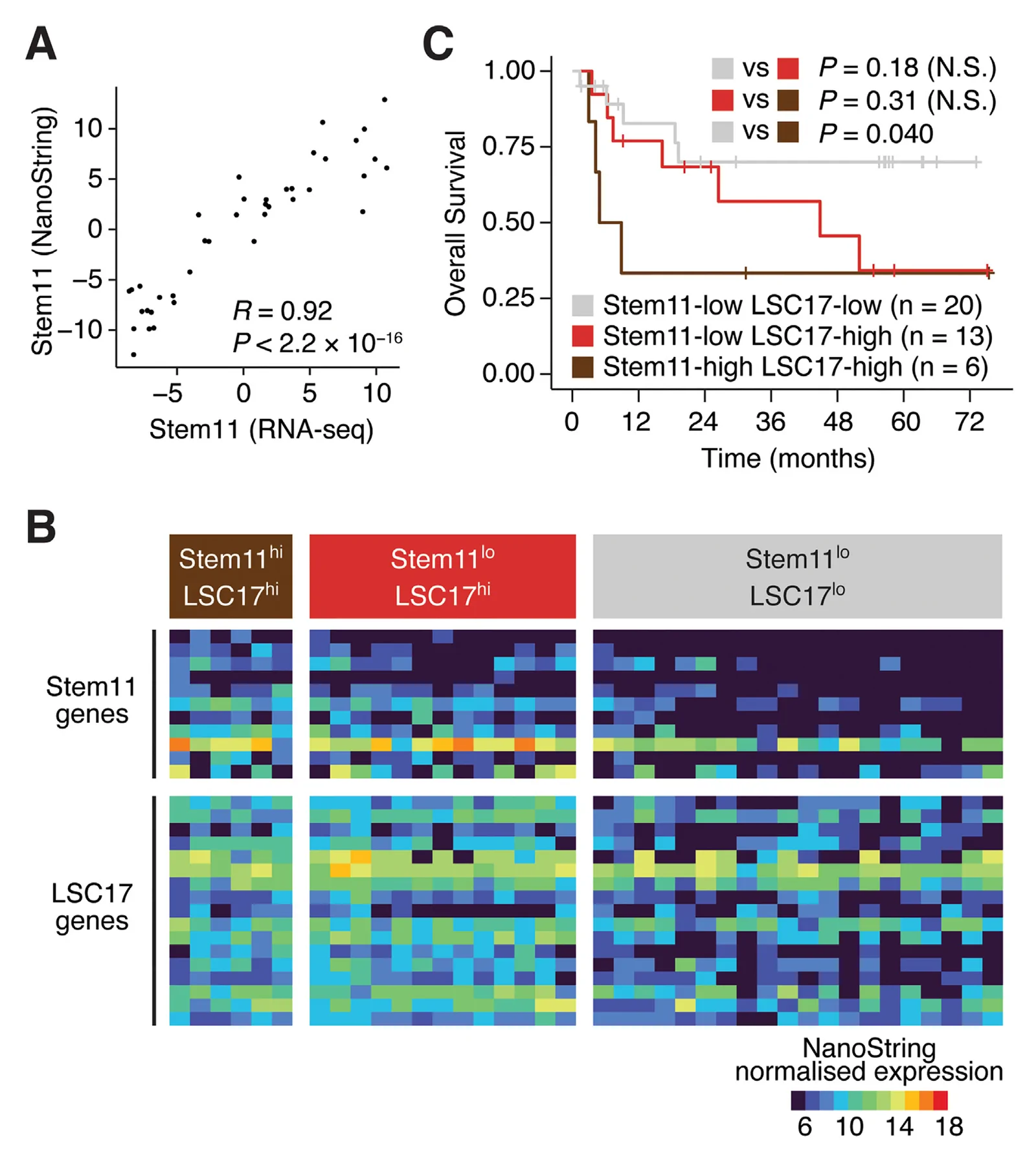
NanoString-based Stem11 classification of AML. **(A)** A scatter plot comparing NanoString-based and RNA sequencing-based Stem11 scores. The Pearson correlation *p-*value is indicated. **(B)** A heatmap showing the normalized expression of Stem11 and LSC17 genes measured using the NanoString custom nCounter panel. Each column represents an individual patient, and rows represent individual genes. **(C)** Survival analysis based on the combined Stem11- and LSC17-based stratification. The *p* values are from the log-rank test.

Although RNA-seq analysis comprehensively validated the prognostic value of the Stem11 scoring system across multiple patient cohorts, its routine diagnostic application is currently impractical due to the substantial time and cost requirements for library preparation and complex data analysis. Therefore, to facilitate potential clinical implementation of the Stem11-based risk stratification, we developed a custom NanoString nCounter panel targeting the Stem11 genes, alongside the previously published LSC17 genes [10] for comparison (Supplementary Table E2). The NanoString nCounter system directly quantifies target messenger RNA (mRNA) molecules using colorcoded probes without the need for amplification or reverse transcription, enabling sensitive and reproducible gene expression measurements from as little as 100 ng of total RNA [17]. The platform offers a standardized workflow with short hands-on time and a built-in analysis pipeline, which could enable results to be returned to clinicians within 48−72 hours [11,17]. To evaluate the performance of this assay, we analyzed 39 de novo AML patients from the same cohort analyzed using RNA-seq in Figure 1. Stem11 scores derived from our NanoString assay showed a strong positive correlation with scores calculated from the RNA-seq data performed on the same samples in the original study [9] (Pearson’s *R* = 0.92, *p* < 2.2 × 10^−16^; Figure 2A), demonstrating the robustness of our NanoString-based Stem11 scoring platform developed in this study. Stem11 and LSC17 classifications were then assigned to each patient, identifying three patient subgroups: Stem11-high LSC17-high, Stem11-low LSC17-high, and Stem11-low LSC17-low (Figure 2B and Supplementary Figure E2A). In our cohort of 39 patients, neither Stem11 nor LSC17 alone achieved significant survival prediction (*p* = 0.066 for Stem11 and 0.072 for LSC17; Supplementary Figure E2B, C), although both showed trends toward poorer survival in high-score patients. Of note, however, their combination revealed significantly poorer outcomes for Stem11-high LSC17-high patients compared with Stem11-low LSC17-low patients (Figure 2C). This survival trend is consistent with RNA-seq-based classification in the original RNA-seq cohort [9] (Supplementary Figure E2D), as well as in the independent Beat AML cohort [13] (Supplementary Figure E2E). Together, these results demonstrate the reliability of our newly developed NanoString-based platform to recapitulate RNA-seq-based Stem11 classification, which has previously been validated across multiple AML cohorts [8,12 −14]. The potential clinical workflow (Supplementary Figure E2F) will integrate with existing diagnostic pipelines, where bone marrow or peripheral blood samples collected at diagnosis would undergo NanoString analysis in parallel with standard cytogenetic and molecular testing. The rapid turnaround time of NanoString assays [11,17] will enable Stem11 classification to be available alongside other diagnostic results, facilitating timely treatment decisions, particularly regarding the consideration of allogeneic HCT.

Finally, several clinical and technical considerations remain before the Stem11-based prognostication can be implemented in routine clinical practice. First, although our RNA-seq cohort comprised more than 100 uniformly treated patients with AML, detailed analyses stratified by the combination of ELN risk groups, Stem11 classification, and HCT status were limited by small subgroup sizes. In our multivariate analysis (Supplementary Figure E1C), the hazard ratio for ELN intermediate-risk patients exceeded that of ELN adverse-risk patients, which may also reflect the limited number of cases and events within each group stratified by ELN classification and HCT status. The precise clinical utility of the Stem11 classification will, there-fore, need to be determined in the future using larger, uniformly treated cohorts. Additionally, the current Stem11 classification relies on *Z*-score-based scoring, which depends on cohort-specific normalization and would therefore need to be adapted for prospective use. Although the current study provides a proof of concept for the Nano-String-based Stem11 scoring system, future clinical implementation will require calibration using a larger cohort analyzed on the same NanoString platform, including technical validation by repeated measurements, to establish a cohort-independent absolute expression threshold with clinical-grade reproducibility. Nevertheless, the unique ability of the Stem11 scoring system to identify patients with particularly poor outcomes, even within each genetic risk group, strongly suggests that further preclinical studies and prospective clinical trials are warranted to enhance current prognostic strategies.

## Supplementary Material

Supplementary material associated with this article can be found in the online version at https://doi.org/10.1016/j.exphem.2026.105441.

Supplementary Figure E

Supplementary Table E

## Data Availability

All raw NanoString data generated in this study are available via ArrayExpress under the accession number E-MTAB-16782.

## Highlights

- Stem11 predicts posttransplantation response and overall survival in acute myeloid leukemia (AML).
- NanoString-derived Stem11 scores show high concordance with RNA sequencing (RNA-seq)-based scores.
- The NanoString Stem11 panel has the potential to enable rapid prognostication for AML.
