# Supplementary material for: Stem11 score: toward rapid clinical prognostication for acute myeloid leukemia
Source: Exp Hematol. Author manuscript; Available in PMC 2026 Oct 16. (PMC7619561; doi:10.1016/j.exphem.2026.105441)
Supplement: Supplementary file 1 [file EMS216239-supplement-Supplementary_Figure_E.pdf]

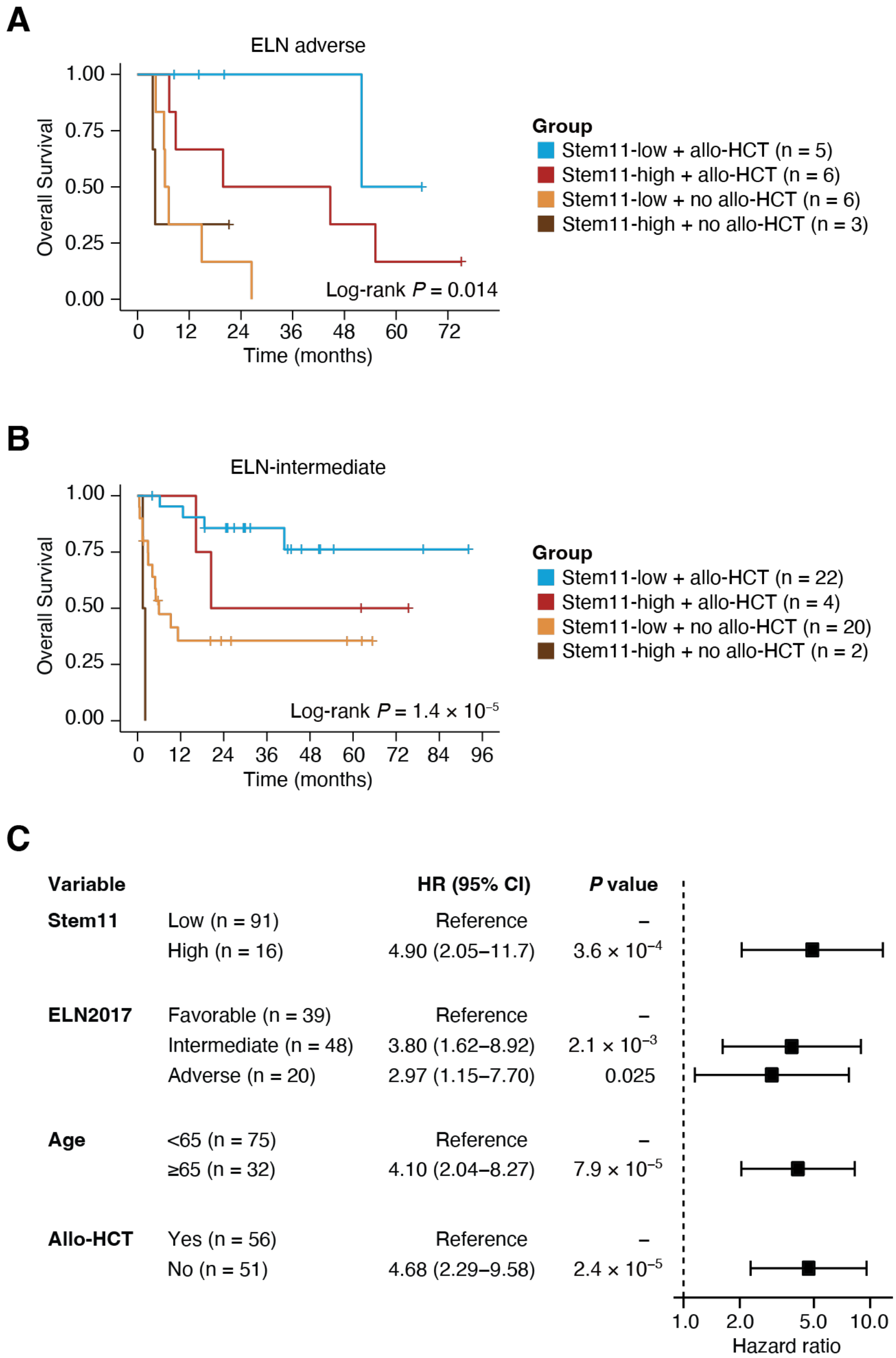

Supplementary Figure E1. Independent prognostic significance of Stem11 in a cohort of uniformly treated AML patients

(A and B) Survival analysis comparing ELN-adverse (A) and ELN-intermediate (B) patients stratified by Stem11 classification and HCT treatment. *P* values are from log-rank test. (C) Multivariate Cox proportional hazards analysis for overall survival in the cohort of uniformly treated AML patients. Stem11 classification, ELN2017 classification, patient age and allogeneic HCT (allo-HCT) were included in the model. Error bars show the 95% confidence interval. HR, hazard ratio; CI, confidence interval.

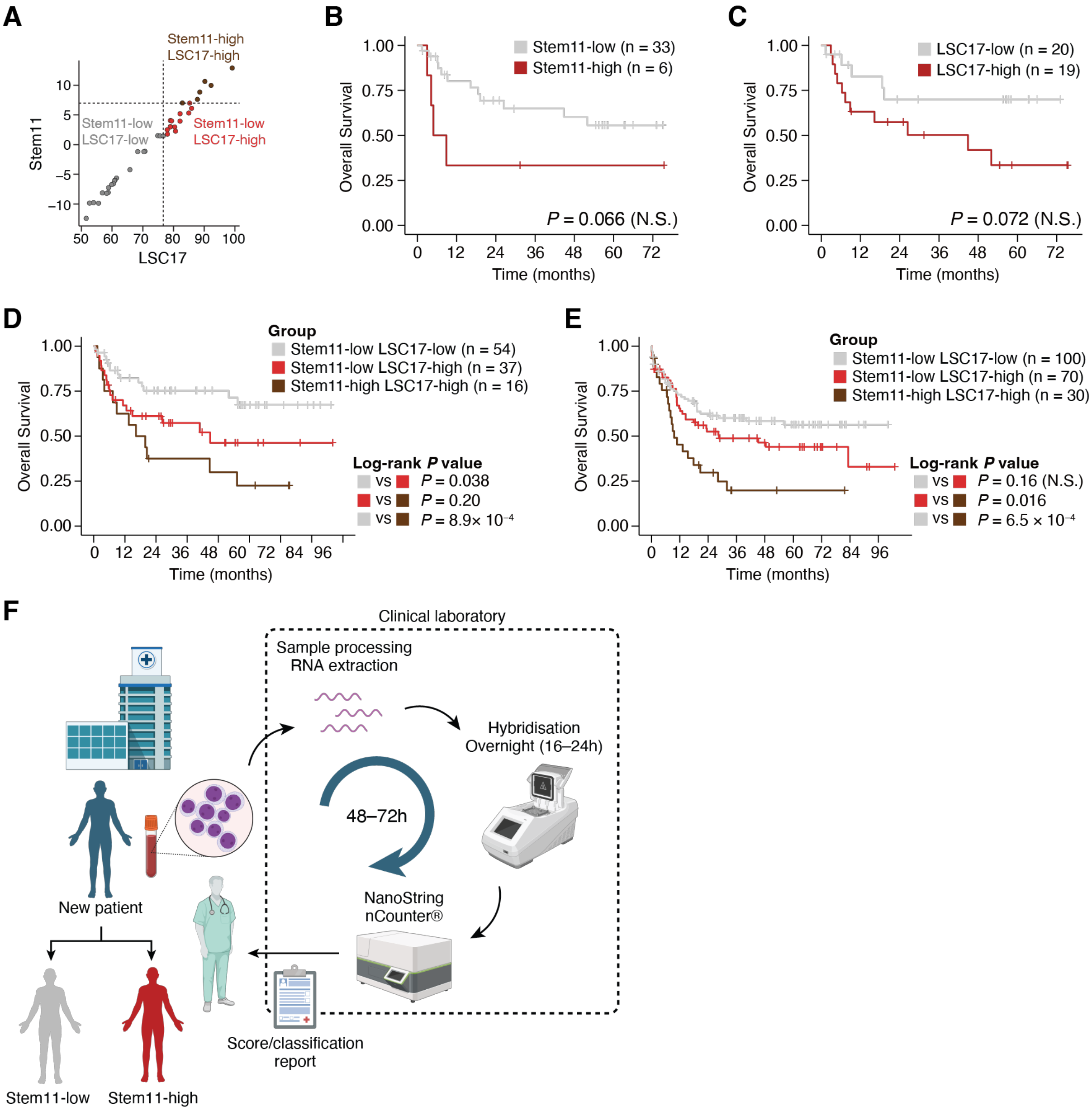

Supplementary Figure E2. Concordant survival predictions based on NanoString and RNA sequencing

(A) A scatter plot showing the Stem11 and LSC17 score distributions. Stem11-high patients were defined as the top 15th percentile of the cohort, as previously defined (ref. 8). LSC17-high patients were defined as those with scores above the cohort median, consistent with the original publication (ref. 10). (B and C) Survival analysis comparing the Stem11-based (B) and LSC17-based (C) stratifications. *P* values are from log-rank test. (D and E) Survival analysis stratified by combined Stem11 and LSC17 risk groups in the German (D) and Beat AML (E) cohorts. N.S., not significant. (F) Schematic diagram illustrating the potential clinical workflow for implementing the NanoString-based rapid Stem11 classification platform for patient risk stratification.
